# Size‐Dependent Habitat Selection in a Tropical Freshwater Crayfish: Preference for Vegetated Refugia

**DOI:** 10.1002/ece3.73540

**Published:** 2026-04-28

**Authors:** Mitchell Heide, Paula Cartwright, Amrit K. Mishra, Antony Squires, Nathan J. Waltham

**Affiliations:** ^1^ Centre for Tropical Water and Aquatic Ecosystem Research, Bebegu Yumba Campus James Cook University Queensland Australia; ^2^ School of Marine Biology and Aquaculture, College of Science and Engineering, Bebegu Yumba Campus James Cook University Queensland Australia

**Keywords:** agricultural development, biodiversity, conservation, turbidity, water quality

## Abstract

Habitat selection is a critical ecological process influencing survival and recruitment during early life stages of freshwater species, yet it remains unclear whether observed habitat use reflects active preference or simple accessibility. We experimentally quantified habitat selection in the freshwater crayfish *Cherax quadricarinatus* across two early life stages (craylings and juveniles), testing whether individuals actively select structurally complex habitats and whether this selection is size‐dependent. Using laboratory choice and no‐choice arenas, we found that both life stages exhibited clear, measurable preferences for vegetated habitats. Craylings selected vegetation 10.7% ± 3.44% more often when choice was available compared with no‐choice conditions, while juveniles showed a markedly stronger response, selecting vegetation 60.0% ± 9.03% more often. In contrast, use of sand declined under choice conditions (craylings: −12.0% ± 4.59%; juveniles: −6.67% ± 15.87%), and gravel showed little or no positive selection (craylings: 2.7% ± 6.64%; juveniles: 0% ± 9.03%). Habitat use in no‐choice arenas was comparatively even, indicating that accessibility alone could not explain observed patterns. Together, these results demonstrate that vegetated habitats provide disproportionately important refuge for early life stages of freshwater crayfish, with juveniles exhibiting five‑ to sixfold stronger selection for vegetation than craylings. Our findings highlight how the loss of habitat complexity through environmental change or anthropogenic disturbance may reduce recruitment success and population viability in tropical freshwater ecosystems.

## Graphical abstract

Young freshwater crayfish need shelter to survive, but it is often unclear whether they actively choose safe habitats or simply remain where they are found. We show that craylings and juvenile red claw crayfish prefer vegetated habitats over bare sand or gravel, and that this preference becomes stronger as crayfish grow. Protecting aquatic vegetation in tropical rivers is therefore important for supporting crayfish populations, particularly during early life stages.

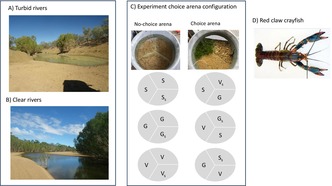

## 1 Introduction

The spatial distribution of animals is rarely random, but instead reflects a series of behavioural decisions that balance access to resources with exposure to risk. Habitat selection is therefore a fundamental ecological process, shaping individual survival, growth and reproductive success and ultimately influencing population and community structure (Wisz et al. 2013; Schmidt et al. 2010). These decisions are especially critical during early life stages, when individuals are most vulnerable to habitat degradation, which in turn could increase susceptibility to predation pressure. Understanding how animals select habitats is now ever more important in freshwater ecosystems, where habitat degradation and biodiversity loss are accelerating globally, with recent assessments indicating that approximately one quarter of freshwater fauna are threatened with extinction (Sayer et al. 2025).

Habitat selection is driven by multiple interacting factors, including food availability, shelter, thermal conditions and predation risk. Many species can assess trade‐offs among these factors to maximise fitness within a given environment (Dall et al. 2012). Structurally complex habitats, such as aquatic vegetation, often provide refugia that reduce predator efficiency while simultaneously enhancing foraging opportunities (Savino and Stein 1989). The strength of habitat selection is frequently size‐dependent, reflecting ontogenetic shifts in vulnerability and energetic requirements. Smaller or juvenile individuals typically show stronger associations with complex habitats than larger conspecifics, a pattern documented across aquatic taxa including fish, crustaceans and amphibians (Schlosser 1987; Persson and Eklov 1995; Amburgey et al. 2014).

Freshwater crayfish are well suited for examining habitat selection because they occupy a wide range of substrates, experience intense predation pressure during early life stages and play key ecological roles as consumers and ecosystem engineers (Reynolds et al. 2013). *Cherax quadricarinatus* , the red claw crayfish, is native to tropical rivers of northern Australia and typically inhabits slow‐flowing or lentic environments characterised by a mosaic of sand, gravel and aquatic vegetation (Baker et al. 2008; Waltham et al. 2013; Wu et al. 2019) – and while its conservation status is not of concern, this species is highly targeted recreationally (Pinder et al. 2019). These habitat elements provide a gradient of structural complexity, ranging from relatively unconsolidated substrates (sand, gravel) to structurally complex refugia (vegetation, woody debris), which are known to influence predator–prey interactions and resource use in freshwater systems.

Following hatching, red claw crayfish progress through early benthic stages (craylings < 15 mm; juveniles 15–50 mm), during which individuals are small, relatively mobile and highly susceptible to predation by fish and larger conspecifics (Garvey et al. 1994; García‐Guerrero et al. 2013). Although general habitat associations of adult crayfish are reasonably well described, far less is known about habitat use and selection during these early life stages, when mortality risk is highest and access to refuge is likely to be a key determinant of survival. Structural habitat features—particularly aquatic vegetation—are widely recognised as important nursery habitats for aquatic fauna because they provide both physical refuge from predators and enhanced foraging opportunities. In contrast, simpler substrates such as sand and gravel may offer limited shelter, potentially increasing exposure to predation.

Northern Australian rivers experience extreme seasonal hydrological variability driven by monsoonal rainfall and prolonged dry periods (Petheram et al. 2012), during which extensive river networks contract into isolated waterholes (Kennard 2010; Warfe et al. 2011). These dry season refugia are increasingly threatened by climate change, agricultural expansion, altered flow regimes and declining water quality (Close et al. 2012; King et al. 2015; Shanafield et al. 2024; Burford et al. 2026). The loss or simplification of aquatic vegetation within these systems may disproportionately affect early life stages of freshwater species by reducing refuge availability and increasing predation risk. However, it remains unclear whether observed associations with habitats reflect active selection or simply the availability of those habitats in the environment. To address this, we experimentally test habitat selection in *Cherax quadricarinatus* using controlled laboratory arenas that isolate preference from accessibility. We focus on three common habitat types—sand, gravel and aquatic vegetation—that represent contrasting levels of structural complexity and are commonly encountered in natural systems. Habitat use is compared across choice and no‐choice configurations and between two early life stages (craylings and juveniles) to determine whether selection is both active and size‐dependent. We hypothesise that crayfish will preferentially select structurally complex, vegetated habitats over simpler substrates, independent of accessibility and that this selectivity will be stronger in juveniles than in craylings due to increasing energetic demands and continued vulnerability to predation. By linking habitat selection to early life‐stage ecology, this study provides mechanistic insight into how habitat degradation may influence recruitment and persistence in tropical freshwater ecosystems.

## 2 Methods

### 2.1 Red Claw Crayfish—Choice Animal

*Cherax quadricarinatus* , commonly known as the red claw crayfish, is a freshwater crustacean native to northern Australia (Baker et al. 2008; James et al. 2017). *C. quadricarinatus* is characterised by its robust, dark body and striking red claws, which are more pronounced in males. These claws serve as tools for communication and defence, with males often engaging in territorial disputes (Karplus et al. 2003). Males can reach sizes of up to 25 cm, whereas females are typically smaller (García‐Guerrero et al. 2013). This species exhibits a wide range of feeding behaviours, being opportunistic omnivores that consume both plant material and animal matter, including detritus, algae and small invertebrates (Giling et al. 2009; Miller et al. 2023). In terms of habitat preferences, this crayfish in the wild is found in slow‐moving rivers, ponds and reservoirs, particularly those with muddy or sandy substrates, which provide ample opportunities for burrowing or accessing areas under fallen timber in rivers or high leaf litter areas, which could be essential for predator avoidance (Wu et al. 2019). Seeking shelter beneath aquatic vegetation also provides an opportunity to thermoregulate, and possibly also avoid predation (Reynolds et al. 2013). Therefore, if these habitat resources were available, the ability to avoid stresses is decreased for this species across its distribution. Red claw craylings and juveniles were sourced from Living Water Aquaculture (Townsville) (where crayfish are reared in tanks with 12 h daytime/12 h nighttime photoperiod and are fed daily), with all experiments completed at James Cook University (JCU), Townsville campus, Australia. Experiments commenced in April and were concluded in July 2024 (ambient air temperature during this period ranged between daily maximum 25°C and 32°C, tank water temperatures ranged between daily maximums of 22°C and 29.5°C—measured using Hobo One Temperature loggers positioned in tanks, and hanging over tanks and programmed to log at 20 min intervals). Two size classes (measured as ocular carapace length) of crayfish were used here: (1) craylings (2.7–4.5 mm total length; 0.011–0.028 g wet weight); and (2) juveniles (35–55 mm, 15.9–45.6 g).

### 2.2 Experimental Tank Configuration

**FIGURE 1:**
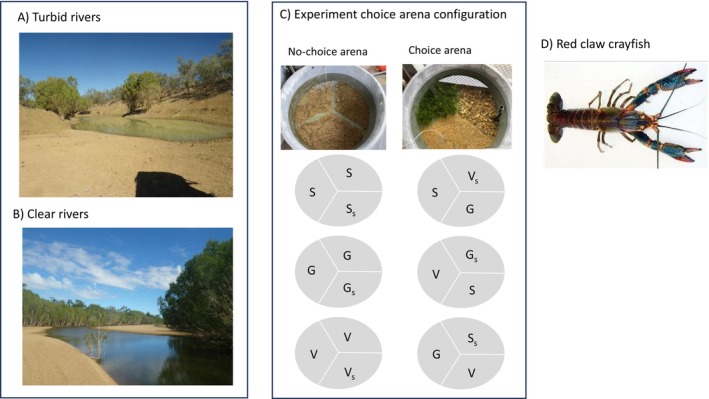
Example of turbid (A—Flinders River, Queensland) and clear (B—Gilbert River, Queensland) waterways in northern Australia. (C) Representation of experiment showing choice and no‐choice arenas, with arenas divided into three sedments; sand (S), gravel (G), and vegetation (V). Habitat starting segments are identified with the subscript s (e.g., G_s_). The same experiment set up was presented to juvenile and adult crayfish. (D) Example of redclaw crayfish.

Circular plastic tanks (H = 0.45 m, diameter = 1.1 m, area = 0.95 m^2^) were filled with aquarium filtered freshwater (~320 L). The tanks were divided into three equal segments (measurement unit), with a habitat, sand (S), gravel (G) and vegetation (V), added to each segment in the choice arenas (Figure 1). A small gap was left between each habitat segment to assist with inserting dividers at the conclusion of the experiment period to prevent crayfish from moving among habitat segments. All habitat materials were collected locally in Townsville. The local sand and gravel creek are similar in particle size to river systems in northern Queensland, and the aquatic vegetation, *Ceratophyllum*, is a widespread emergent plant species found in clear water systems in northern Queensland (Kerrigan et al. 2011). The *Ceratophyllum* was wrapped around larger stones (2–4 pieces ~10 cm long) and placed into the choice arena to provide a dense vegetation choice for crayfish (see Figure 1).

Three choice arenas were set up in an outdoor laboratory compound at JCU (with natural light with a photoperiod of 12 h daylight and 12 h nightlight), that has a roof and open sides. Choice arenas were set up and allowed to settle over 3 days prior to the experiment commencing. The no‐choice arenas were set up in a similar way, though each segment had the same habitat (Figure 1). At the commencement of the choice and no‐choice experiment, after the tank environment had settled, animals were added and were free to roam for 3 days before the dividers were inserted, and the arenas drained by pumping the water out using an aquarium hose. The water was passed through a 2 mm sieve before discharging to the aquarium recirculatory system to ensure that juvenile crayfish could not be lost from the experiment during this phase. Once all the water had been removed, and the habitats from each segment thoroughly searched for crayfish (for ~30 min each segment). The proportion (total of 50 individuals in each experiment) of craylings and juveniles recovered within each segment was calculated by dividing by the total individuals recovered from each arena. Experiments were repeated three times, for each combination of choice and no‐choice arenas, where each time new crayfish (craylings and juveniles) were sourced from our main supply tank (crayfish at the end of each experiment were stored in separate recover tanks from the main tank, so no crayfish was used twice in an experiment).

### 2.3 Experimental Design and Analysis

The approach here to place animals within a single habitat segment was necessary to examine the extent to which crayfish remain in a segment or leave in the presence or absence of choice. In this experiment, a crayfish was considered to have made a choice to select vegetation if the proportion found in the vegetation segment was greater than would have been predicated from their distribution in the absence of choice—comparable to Webley et al. (2009). The experiment had two factors (choice, two levels—choice and no‐choice) and habitat start segment (three levels—start in sand, gravel or vegetation). The data were arc‐sine transformed to account for the nature of the data as proportions. Data were checked for heterogeneity of variance (Cochran's test) before using an ANOVA for the selectivity hypothesis was tested, with Choice × Habitat Start Segment significant interaction supporting selectivity. Finally, a Student–Newman–Keuls (SNK) test was completed following significant interactions to determine which habitats were selected by crayfish.

To determine if there was a significant difference in the recovery of animals from different habitats, animal numbers recovered from the no‐choice arena were compared using ANOVA (single factor: Habitat, 3 levels, *n* = 3). Further, similar to Webley et al. (2009), to determine whether animals had sufficient time and were able to access the entire arena, the distribution of crayfish among the different segments of the no‐choice arenas was analysed, using an ANOVA on arc‐sine transformed data testing the factors (Habitat, three levels; segments, three levels fixed), and their interaction.

## 3 Results

### 3.1 Crayfish Return Rate

**FIGURE 2:**
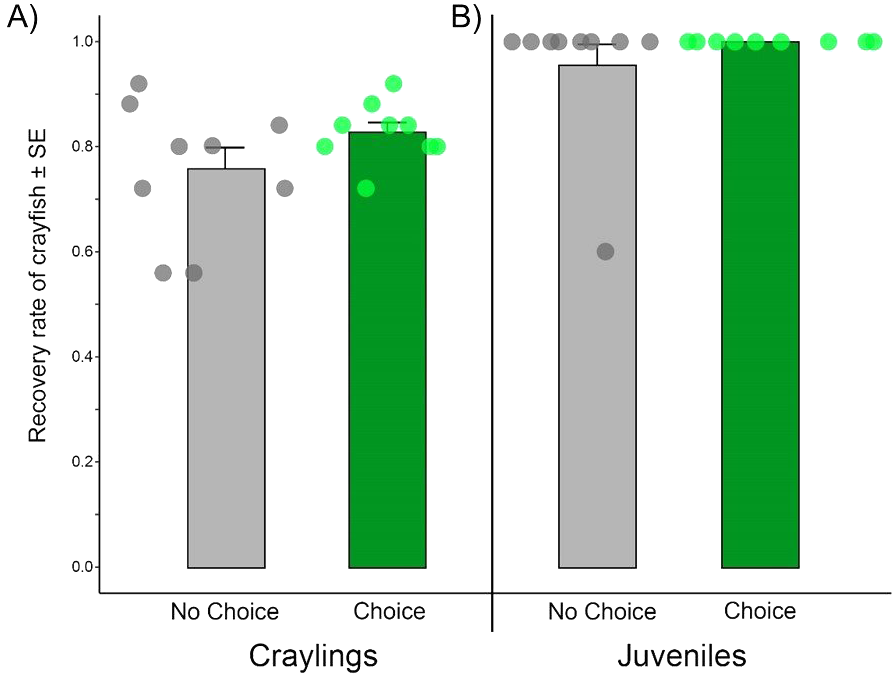
Proportion of starting crayfish return at the conclusion of the experiment for: (A) Craylings; and (B) juvenile individuals for both choice and no‐choice arenas. Mean (± SE) values for the three replicates for each treatment.

After 3 days in the experiment, the return percentage of crayfish (both craylings and juveniles) was between 75% and 100%, regardless of choice or no‐choice configuration (Figure 2). Fewer craylings were retrieved after the experiment, presumably given the smaller size compared with juveniles, where nearly all were retrieved.

### 3.2 Habitat Preferences

**FIGURE 3:**
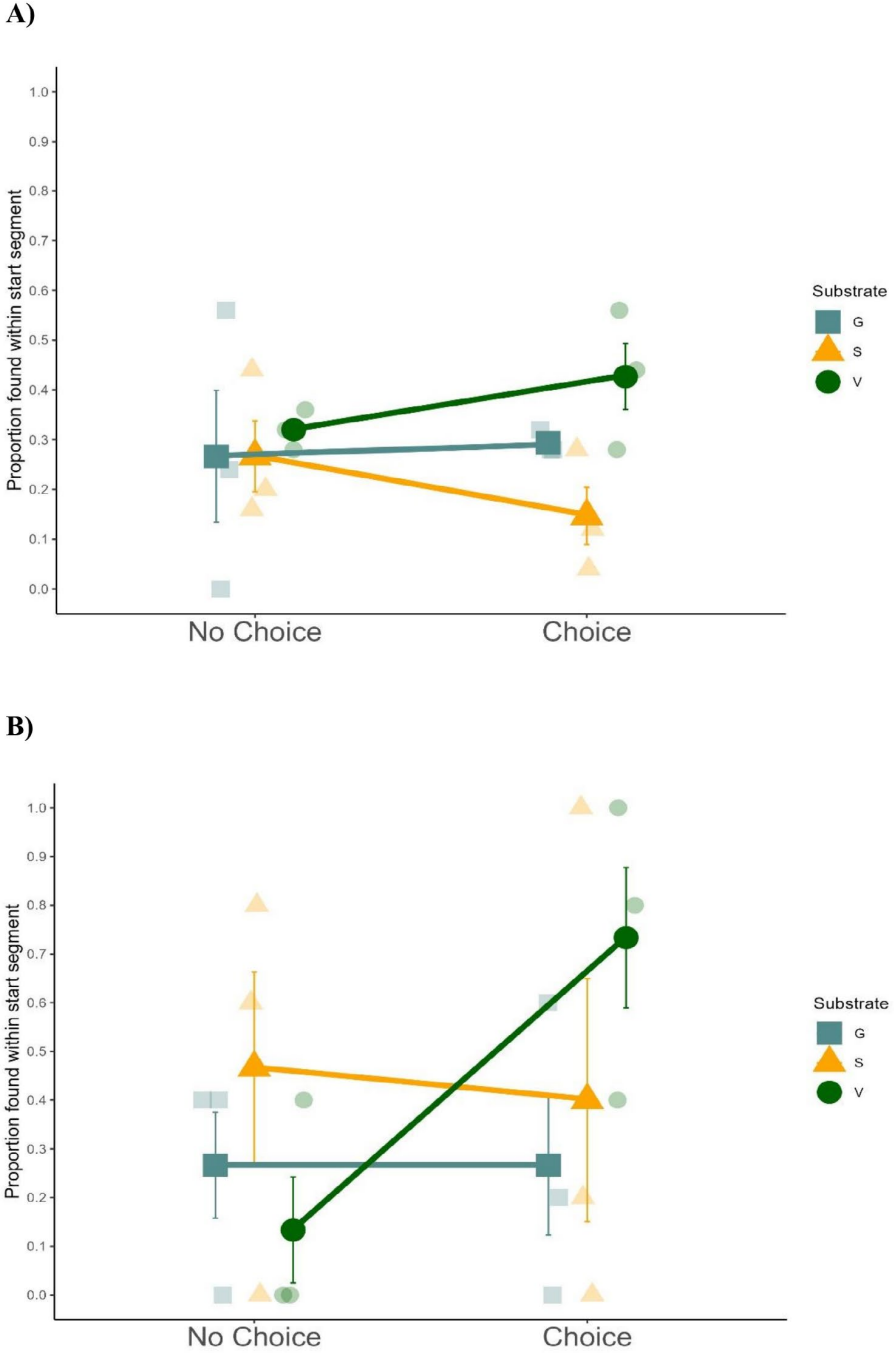
The interaction between the presence or absence of choice and the starting habitat segment for: (A) Craylings and; (B) juveniles is presented. The data represent the mean (SE) of the number of animals found within the starting segment, as a proportion of the total animals recovered from each arena, for the different habitat types in both no‐choice and choice arenas.

Craylings displayed a habitat preference, with significance for substrate type and the interaction of choice and substrate (Table S1). When given a choice of the three habitats, they were found in vegetation 10.7% ± 3.44% more often than with no‐choice (calculation: Choice proportion‐no‐choice proportion). Gravel had a difference of 2.7% ± 6.64% with choice, and sand had—12.0% ± 4.59% difference when choice was available (Figure 3A). Tukey's HSD post hoc test indicated a significant difference in proportions between vegetation and sand (Table S2), while the SNK test displayed significant differences between all three substrates (Table S3). The SNK test also supported that the proportion of craylings in vegetation was significantly higher when choice is available, and the proportion in sand is significantly lower when choice is available (Table S4).

Juveniles showed preferences for vegetation, and less of a preference for sand or gravel. When juveniles had a habitat choice, vegetation had a difference of 60.0% ± 9.03%, gravel had 0% ± 9.03% and sand −6.67% ± 15.87% (Figure 3B). An ANOVA was completed for choice and substrate which found no significant results (Table S5). However, the ANOVA for position and substrate showed significance for substrate in juveniles, with vegetation having a significantly larger proportion than the other substrates (Table S6). Both gravel and sand each have overlapping error with zero difference between no choice and choice, which could mean there is no preference at all.

### 3.3 Habitat Choice—Starting Habitat

**FIGURE 4:**
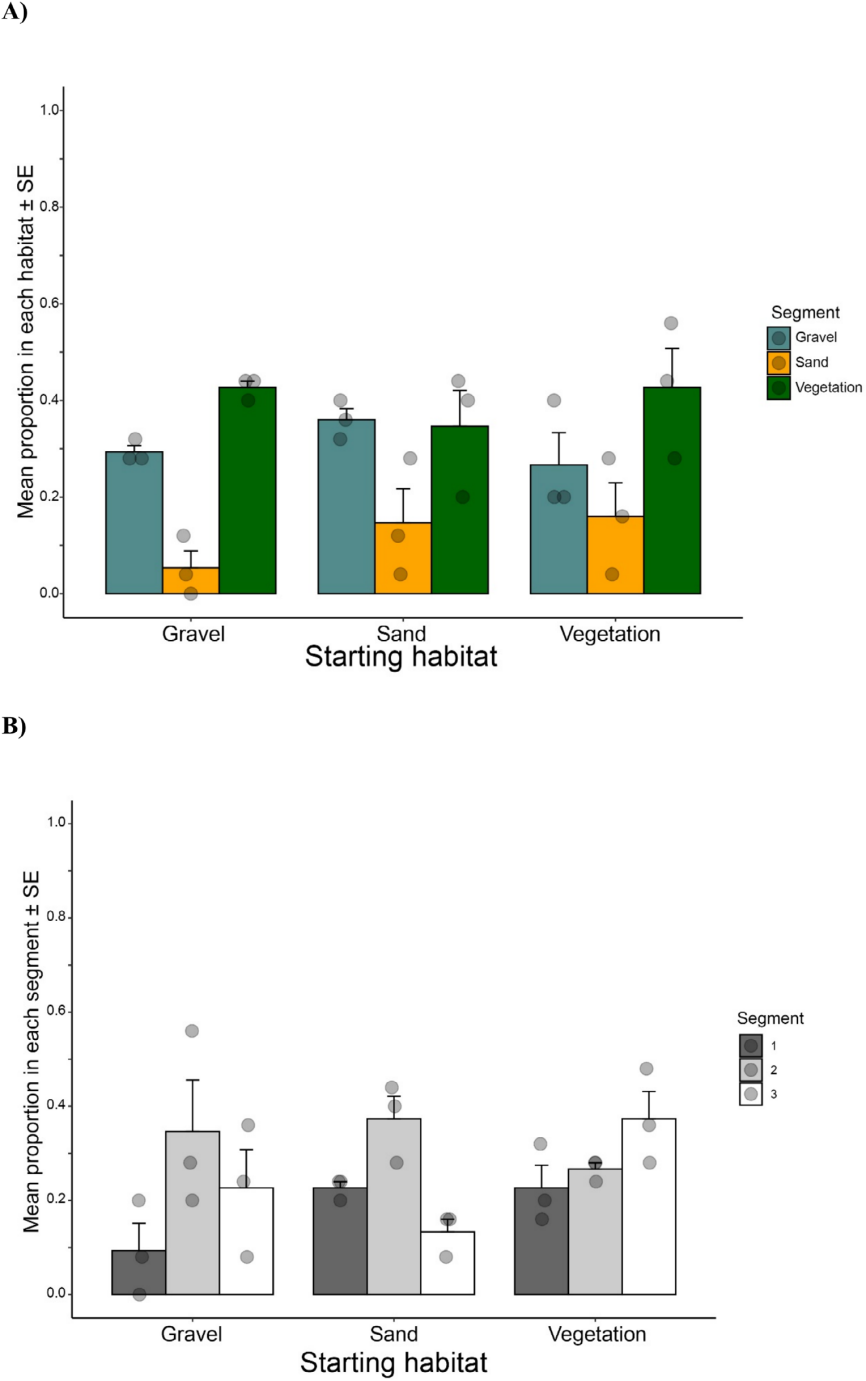
Proportion of craylings found in segments: (A) when in starting habitat segment in Choice areana experiments (Vegetation, green bars; Gravel, blue bars; Sand, yellow bars); and (B) when in no‐choice habitat areans (Starting segment, black bar; Segiment 1, grey bar; Segiment 2, white bar). Mean (± SE) values for three replicates for each treatment.

The proportion of craylings in the starting habitat varies when given a choice. Gravel and vegetation both have increases in proportion, at around 40% respectively. Sand only has about 30% of the original crayfish. This suggests the craylings are seeking vegetation and gravel, over sand, when given the choice (Figure 4A). If they started in gravel, many stayed, but more migrated to vegetation compared with sand. With vegetation as the starting habitat, most stayed, some moved to gravel while only a few moved to sand. In the no‐choice arenas, craylings appear to have no preference for starting segments (Figure 4B).

**FIGURE 5:**
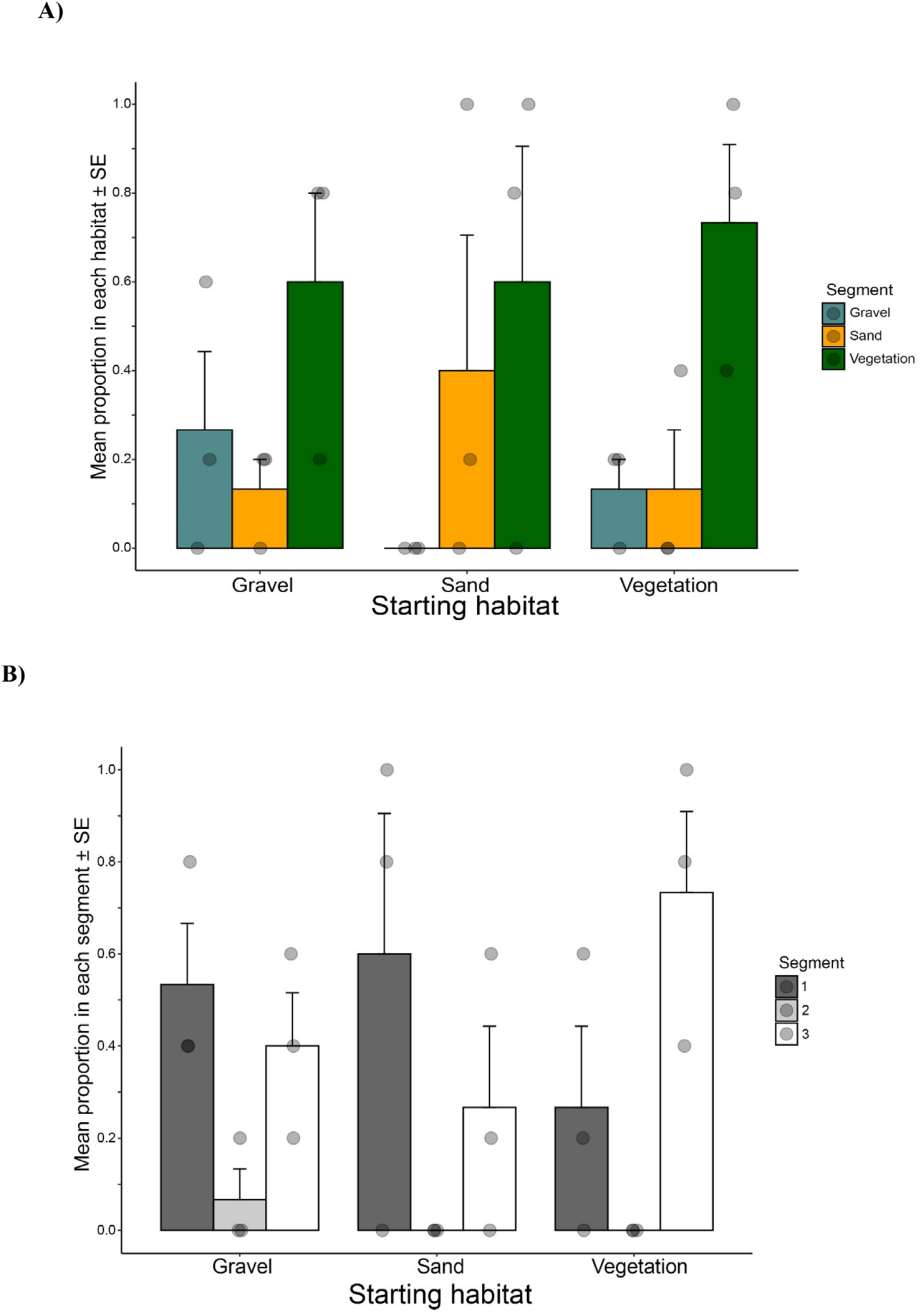
Proportion of juveniles found in segments: (A) when in starting habitat segment in Choice areana experiments (Vegetation, green bars; Gravel, blue bars; Sand, yellow bars); and (B) when in no‐choice habitat areans (Starting segment, black bar; Segiment 1, grey bar; Segiment 2, white bar). Mean (± SE) values for three replicates for each treatment.

In comparison, the proportion of juveniles in the starting habitat also varied when given a choice. Gravel and vegetation both had increases in proportion, up to 70% for vegetation (Figure 5A). Interestingly, when starting in sand, most either stayed in sand or moved to gravel, with only a small number moving to vegetation. In the no‐choice arenas, juveniles appear to have preferred segments 1 and 3, with fewer in segment 2 (Figure 5B). ANOVAs were completed for craylings and juveniles for choice, starting substrate and the interactions. No significance was found for any categories of craylings (Table S7), but there was for juveniles (Table S8).

## 4 Discussion

The decision‐making processes that govern animal movement are not only important for individual survival but have broader ecological and evolutionary implications. In making decisions about when to move for the basic reason to just survive, animals contribute to the structuring of populations and communities in ecosystems. Our laboratory findings provide evidence that craylings, but particularly so juvenile crayfish, exhibit a preference for vegetated habitats when presented the choice or had no choice, compared with sand or gravel substrates, thereby supporting our hypothesis that habitat selection is influenced by the interplay between size and vulnerability to predation. This size‐dependent association in habitat selection aligns with observations in various aquatic and terrestrial species. For instance, a study on perch ( *Perca fluviatilis* ) found that size‐dependent habitat use and size‐specific predation risk, particularly from cannibalistic conspecifics, influence the timing of habitat shifts in young‐of‐the‐year perch (Persson and Eklov 1995). Similarly, research on blue crabs ( *Callinectes sapidus* ) demonstrated that larger individuals may develop stronger intrinsic habitat preferences for complex structures like kelp, possibly due to increased exposure and learned behaviours favouring such environments (Micheli 1997). This animal behaviour has been also observed in terrestrial ecosystems, where body size was shown to influence the scale at which animals interact with their environment, with larger species often selecting habitats that accommodate their greater spatial requirements and movement capabilities (Haskell et al. 2002). This size‐dependent habitat selection highlights the critical importance of vegetated environments, in this case tropical river systems of northern Australia, where this species is found, for early life stages of crayfish but particularly so for juveniles (García‐Guerrero et al. 2013).

The functional role of vegetated habitats presumably is in the cover it offers from predators, but also for the abundant foraging opportunities it creates, which are both crucial for the survival and growth of smaller individuals (Heck and Crowder 1991). Several studies on aquatic and terrestrial animals have demonstrated that habitat preference shifts with size, likely due to differences in vulnerability to predators and the availability of suitable refuge. For example, juvenile freshwater fish such as salmonids show a tendency to favour complex habitats, such as vegetated areas, to avoid predation (Simenstad and Cordell 2000). As these fish grow, they may shift to more open or less structured habitats, as their size provides larger protection from predators (Schlosser 1987). Similarly, the shift in habitat preference with size has been documented in terrestrial animals such as amphibians. Larger amphibians (Amburgey et al. 2014; Graeter et al. 2008), such as bullfrogs ( *Lithobates catesbeianus* ), tend to occupy deeper water or open areas as they grow, avoiding the more complex, predator‐proof habitats preferred by their smaller counterparts (Cushman 2006).

With the well‐established role of vegetated areas as critical nursery habitats for many aquatic species, their loss in the Gilbert River catchment may mean increased exposure pressure to juveniles and craylings to high order consumers. For instance, Savino and Stein (1989) demonstrated that the presence of vegetation reduced the predation risk for juvenile fish by providing structural complexity, which interferes with predator efficiency. Similarly, Dibble et al. (1997) emphasised the role of aquatic vegetation in supporting higher densities of juvenile fish and invertebrates, highlighting its importance for species during their early life phases. In northern Australia's tropical river systems, the seasonal transformation of expansive rivers into a series of diminishing waterholes during dry periods significantly impacts the survival strategies of aquatic organisms (Douglas et al. 2005; Close et al. 2012; Kennard 2010; Rayner et al. 2009). As these waterholes become shallower and more isolated (Jardine et al. 2012; McJannet et al. 2014), craylings (juvenile crayfish) might face heightened predation risks from larger fish due to reduced spatial refuges that are known to consume freshwater crayfish (Pusey et al. 2007). Access to vegetated habitats during these early life stages is therefore crucial, as such environments offer essential cover and foraging opportunities and enhancing survival rates. Studies have shown that many crayfish species require heterogeneous habitats with ample refuges to support various life stages, underscoring the importance of complex structures like submerged vegetation in providing necessary shelter and resources (Beatty et al. 2005; Cortés‐Jacinto et al. 2004; Garvey et al. 1994). The ecological dynamics of waterholes are pivotal in determining recruitment success for both fish and crustaceans (Arthington et al. 2005; Balcombe et al. 2015; Wallace et al. 2017). Research indicates that water level fluctuations can significantly affect fish reproductive success by altering habitats, food availability and exposure to predators (Pettit et al. 2012). For instance, variations in water levels impact littoral zones, which are critical for fish spawning and juvenile development. Similarly, in intermittent dryland rivers, the movement behaviour of fish from isolated waterhole refugia during connecting flow events is crucial for maintaining population connectivity and resilience (Agostinho et al. 2009; Hurd et al. 2016; Jardine et al. 2012). These findings suggest that maintaining habitat complexity within waterholes is essential for supporting the early life stages of aquatic species. Our study aligns with these observations, supporting the model of preference to habitat as an adaptive response to the increased predation pressures associated with contracting waterholes. The degradation or loss of such vegetated areas, whether through natural processes or anthropogenic activities, could therefore have profound implications for crayfish recruitment and overall population viability. Effective management of tropical river catchments should prioritise the preservation and restoration of vegetated habitats within waterholes, ensuring that these critical refuges remain available during periods of environmental stress, like thermal relief for fish and crustaceans (Wallace et al. 2017; Waltham 2018).

Some caution is necessary with the results as the crayfish were sourced from an aquaculture facility, where individuals could have an inherent cognitive risk advert awareness to predation given there are generally no predators to consume species in aquaculture. This may influence their habitat preferences compared with wild counterparts that may have developed cognitive adaptations to navigate diverse habitats during early life stages, enhancing their survival (Budaev et al. 2019). Consequently, the habitat preferences observed in our study could be influenced by the origin of the crayfish, highlighting the importance of considering the source of study organisms when interpreting behavioural data.

Animals are rarely randomly distributed through the environment. Their movements and decisions regarding habitat selection are influenced by a range of cognitive processes, such as spatial memory, risk assessment and learned behaviours. These decisions are not only important for individual fitness but also play a role in the ecological and evolutionary dynamics of freshwater species (Dudgeon et al. 2006). As our understanding of the cognitive mechanisms behind animal movement deepens, it offers valuable insights for conservation managers seeking to protect and manage wildlife populations in an increasingly fragmented and changing world. In northern Australia, where aquatic vegetation is removed or lost, either following major changes in water quality or drying out following over extraction, the result may be a reduction in recruitment success and population viability.

## Author Contributions

**Mitchell Heide:** data curation (equal), formal analysis (equal), investigation (equal), methodology (equal), resources (equal), validation (equal), writing – original draft (equal), writing – review and editing (equal). **Paula Cartwright:** investigation (equal), supervision (equal), writing – review and editing (equal). **Amrit K. Mishra:** data curation (equal), supervision (equal), writing – original draft (equal). **Antony Squires:** investigation (equal), methodology (equal), writing – original draft (equal). **Nathan J. Waltham:** conceptualization (equal), funding acquisition (equal), resources (equal), supervision (equal), validation (equal), visualization (equal), writing – original draft (equal), writing – review and editing (equal).

## Funding

This work was supported by the Australian Government.

## Conflicts of Interest

The authors declare no conflicts of interest.

## Supporting information


**Table S1:** ANOVA test for the comparison between choice and substrate for craylings in the experimental arenas. Significant codes: 0 ‘***’ 0.001 ‘**’ 0.01 ‘*’ 0.05 ‘.’ 0.1.
**Table S2:** Tukey's HSD to identify which substrates had significant differences in proportion based on choice for craylings only in the experimental arenas. Significant codes: 0 ‘***’ 0.001 ‘**’ 0.01 ‘*’ 0.05 ‘.’ 0.1.
**Table S3:** SNK test (*a* = 0.05) to identify which substrates had significant differences in proportion from each other for craylings in the experimental arenas. Groups with the same letter do not have significantly different proportions.
**Table S4:** SNK tests to identify significant substrate levels and directions of proportions based on choice for craylings in experimental arenas. Significant codes: 0 ‘***’ 0.001 ‘**’ 0.01 ‘*’ 0.05 ‘.’ 0.1.
**Table S5:** ANOVA test for the comparison between choice and substrate for juveniles in the experimental arenas. Significant codes: 0 ‘***’ 0.001 ‘**’ 0.01 ‘*’ 0.05 ‘.’ 0.1.
**Table S6:** SNK test (*a* = 0.05) to identify which Substrates had significant differences in proportion from each other. This is for juveniles only. Groups with the same letter do not have significantly different proportions.
**Table S7:** ANOVA comparison for between choice and starting substrate for craylings in experimental arenas. Significant codes: 0 ‘***’ 0.001 ‘**’ 0.01 ‘*’ 0.05 ‘.’ 0.1.
**Table S8:** ANOVA comparison for between choice and starting substrate for juveniles in experimental arenas. Significant codes: 0 ‘***’ 0.001 ‘**’ 0.01 ‘*’ 0.05 ‘.’ 0.1.

## Data Availability

Data can be accessed here https://doi.org/10.25903/88xx‐9g32.
