# Supplementary material for: Size‐Dependent Habitat Selection in a Tropical Freshwater Crayfish: Preference for Vegetated Refugia
Source: Ecol Evol. 2026 Apr 28;16(5):e73540. doi: 10.1002/ece3.73540 (PMC13122256; doi:10.1002/ece3.73540)
Supplement: Supplementary file 1 — Table S1: ANOVA test for the comparison between choice and substrate for craylings in the experimental arenas. Significant codes: 0 ‘***’ 0.001 ‘**’ 0.01 ‘*’ 0.05 ‘.’ 0.1. Table S2: Tukey's HSD to identify which substrates had significant differences in proportion based on choice for craylings only in the experimental arenas. Significant codes: 0 ‘***’ 0.001 ‘**’ 0.01 ‘*’ 0.05 ‘.’ 0.1. Table S3: SNK test (a = 0.05) to identify which substrates had significant differences in proportion from each other for craylings in the experimental arenas. Groups with the same letter do not have significantly different proportions. Table S4: SNK tests to identify significant substrate levels and directions of proportions based on choice for craylings in experimental arenas. Significant codes: 0 ‘***’ 0.001 ‘**’ 0.01 ‘*’ 0.05 ‘.’ 0.1. Table S5: ANOVA test for the comparison between choice and substrate for juveniles in the experimental arenas. Significant codes: 0 ‘***’ 0.001 ‘**’ 0.01 ‘*’ 0.05 ‘.’ 0.1. Table S6: SNK test (a = 0.05) to identify which Substrates had significant differences in proportion from each other. This is for juveniles only. Groups with the same letter do not have significantly different proportions. Table S7: ANOVA comparison for between choice and starting substrate for craylings in experimental arenas. Significant codes: 0 ‘***’ 0.001 ‘**’ 0.01 ‘*’ 0.05 ‘.’ 0.1. Table S8: ANOVA comparison for between choice and starting substrate for juveniles in experimental arenas. Significant codes: 0 ‘***’ 0.001 ‘**’ 0.01 ‘*’ 0.05 ‘.’ 0.1. [file ECE3-16-e73540-s001.docx]

**Supplementary**

Table S1. ANOVA test for the comparison between Choice and Substrate for craylings in the experimental arenas. Significant codes: 0 ‘***’ 0.001 ‘**’ 0.01 ‘*’ 0.05 ‘.’ 0.1

|  | *df* | F | P |  |
| --- | --- | --- | --- | --- |
| Choice | 1 | 0.230 | 0.6334 |  |
| Substrate | 2 | 8.610 | 0.0006 | *** |
| Choice:Substrate | 2 | 6.497 | 0.0032 | ** |
| Residuals | 48 |  |  |  |

Table S2. Tukey’s HSD to identify which Substrates had significant differences in proportion based on Choice for craylings only in the experimental arenas. Significant codes: 0 ‘***’ 0.001 ‘**’ 0.01 ‘*’ 0.05 ‘.’ 0.1.

| Substrate Interactions | Difference | P |  |
| --- | --- | --- | --- |
| S-G | -0.1063 | 0.10302 |  |
| V-G | 0.1050 | 0.10876 |  |
| V-S | 0.2113 | 0.00039 | *** |

Table S3. SNK test (a=0.05) to identify which substrates had significant differences in proportion from each other for craylings in the experimental arenas. Groups with the same letter do not have significantly different proportions.

| Substrate | *Proportion* | Groups |
| --- | --- | --- |
| Vegetation | 0.6226 | a |
| Gravel | 0.5176 | b |
| Sand | 0.4113 | c |

Table S4. SNK tests to identify significant Substrate levels and directions of proportions based on Choice for craylings in experimental arenas. Significant codes: 0 ‘***’ 0.001 ‘**’ 0.01 ‘*’ 0.05 ‘.’ 0.1.

| Substrate | *P* | Direction |
| --- | --- | --- |
| Vegetation | >0.05 | Choice > No Choice |
| Gravel | NS | NA |
| Sand | >0.05 | Choice < No Choice |

Table S5. ANOVA test for the comparison between Choice and Substrate for juveniles in the experimental arenas. Significant codes: 0 ‘***’ 0.001 ‘**’ 0.01 ‘*’ 0.05 ‘.’ 0.1

|  | *df* | F | P |  |
| --- | --- | --- | --- | --- |
| Choice | 1 | 0.065 | 0.7995 |  |
| Substrate | 2 | 2.712 | 0.0766 | . |
| Choice:Substrate | 2 | 2.638 | 0.0818 | . |
| Residuals | 48 |  |  |  |

Table S6. SNK test (a=0.05) to identify which Substrates had significant differences in proportion from each other. This is for juveniles only. Groups with the same letter do not have significantly different proportions.

| Substrate | *Proportion* | Groups |  |
| --- | --- | --- | --- |
| Vegetation | 0.7462 | a |  |
| Gravel | 0.4029 | b |  |
| Sand | 0.4263 | b |  |

Table S7. ANOVA comparison for between Choice and Starting Substrate for craylings in experimental arenas. Significant codes: 0 ‘***’ 0.001 ‘**’ 0.01 ‘*’ 0.05 ‘.’ 0.1.

|  | df | F | P |
| --- | --- | --- | --- |
| Choice | 1 | 0.148 | 0.703 |
| StartingSubstrate | 2 | 0.908 | 0.410 |
| Choice:StartingSubstrate | 2 | 0.131 | 0.877 |
| Residuals | 48 |  |  |

Table S8. ANOVA comparison for between Choice and Starting Substrate for juveniles in experimental arenas. Significant codes: 0 ‘***’ 0.001 ‘**’ 0.01 ‘*’ 0.05 ‘.’ 0.1.

|  | df | F | P |
| --- | --- | --- | --- |
| Choice | 1 | 0.054 | 0.818 |
| StartingSubstrate | 2 | 2.074 | 0.049* |
| Choice:StartingSubstrate | 2 | 0.023 | 0.977 |
| Residuals | 48 |  |  |
